# Contemporary use of arterial and venous conduits in coronary artery bypass grafting: anatomical, functional and clinical aspects

**DOI:** 10.1007/s12471-016-0919-2

**Published:** 2016-11-22

**Authors:** G. Cuminetti, S. Gelsomino, S. Curello, R. Lorusso, J. G. Maessen, J. C. A. Hoorntje

**Affiliations:** 1 Department of Cardiology, Maastricht University Medical Center, Maastricht, The Netherlands; 2 Department of Cardiac Surgery, Maastricht University Medical Center, Maastricht, The Netherlands; 3 Spedali Civili and University of Brescia, Brescia, Italy

**Keywords:** Graft selection, Coronary artery bypass grafting, Myocardial revascularisation, Hybrid coronary artery revascularisation

## Abstract

Although the benefits of using the left internal mammary artery to bypass the left anterior descending artery (LAD) have been extensively ascertained, freedom from major cardiovascular events and survival after coronary artery bypass grafting (CABG) also correlate with the completeness of revascularisation. Hence, careful selection of the second-best graft conduit is crucial for CABG success. The more widespread use of saphenous vein grafts contrasts with the well-known long-term efficacy of multiple arterial grafting, which struggles to emerge as the procedure of choice due to concerns over increased technical difficulties and higher risk of postoperative complications. Conduit choice is at the discretion of the operator instead of being discussed by the heart team, where cardiologists are not usually engaged in such decisions due to a hypothetical lack of technical knowledge. Furthermore, according to the ESC/EACTS guidelines, traditional CABG remains the gold standard for multi-vessel coronary artery disease with complex LAD stenosis, but hybrid procedures using percutaneous coronary intervention for non-LAD targets could combine the best of two worlds. With the aim of raising the cardiologist’s awareness of the surgical treatment options, we provide a comprehensive overview of the anatomical, functional and clinical aspects guiding the decision-making process in CABG strategy.

## Introduction

The choice of the optimal revascularisation strategy in patients with multi-vessel disease has been a great challenge for worldwide interventional cardiologists and surgeons for a long time. In the last 15 years many large-scale randomised trials have compared multi-vessel percutaneous coronary intervention (PCI) with coronary artery bypass grafting (CABG) in terms of long-term survival rate, treatment efficacy and incidence of major adverse cardiovascular events (MACE) [1–3].

Lately, technological progress and new findings in pharmacological therapy have led to a significant improvement in clinical outcomes following PCI. The use of new-generation drug-eluting stents (DES), glycoprotein IIb/IIIa receptor inhibitors and bivalirudin produced an undoubted reduction in restenosis, ischaemic and bleeding complication rates [4–8]. Furthermore, intensive medical management and widespread implementation of cardiovascular prevention have led to a better control of coronary artery disease (CAD) progression.

Despite such significant improvements, European Society of Cardiology/European Association for Cardio-Thoracic Surgery (ESC/EACTS) guidelines still recommend patients with less extensive CAD be treated with PCI, while those with left main or three-vessel disease, particularly when the proximal left anterior descending artery (LAD) is involved, be diverted to CABG [9].

The selection of the graft conduit is influenced by various factors and is crucial for CABG success, affecting survival, freedom from myocardial infarction, symptoms and re-interventions, and correlating with patient outcome [10].

The choice of conduit for CABG seems to be still at the discretion of the operator instead of being discussed by the heart team, and this is probably why specific guidelines have been recently published on this subject [11]. Therefore, although cardiologists should have an important role in selecting the most appropriate conduits together with the surgeon, they are usually not deeply involved due to a hypothetical lack of technical knowledge.

With the aim of raising the cardiologist’s awareness of the surgical treatment options, we provide a comprehensive overview of the anatomical, functional and clinical aspects guiding the decision-making process in CABG strategy.

## Conduit classification

The first choice that operators are called to make is whether to use a venous or an arterial conduit. In general, the main advantage of arterial grafts is their superior long-term patency compared with saphenous vein grafts (SVGs) and, accordingly, arterial grafts are more indicated in younger patients or in those who have a life expectancy of more than 10 years, which is beyond the benefit of SVGs [12–14]. On the other hand, the technique of arterial grafting is more challenging and time-consuming, and therefore venous grafting is preferred in emergency situations and for patients with a higher operative risk.

While vein grafts act merely as conduits, arterial grafts have the ability to adapt to different demands of blood supply and show specific functional properties that will be subsequently discussed. The structure of the arteries differs in elastic and muscular composition, thus some are more reactive to vasoconstrictors than others. From this assumption, derived from extensive experiments on vasoreactivity, a functional classification for arterial grafts into three subtypes was proposed by He and Yang [15]: type I, somatic arteries; type II, splanchnic arteries; and type III, limb arteries. Due to the higher degree of smooth muscle cells over elastic fibres, type II and III arteries show higher contractility and are prone to spasm [15, 16].

## Left internal mammary artery

It has long been accepted that using the left internal mammary artery (LIMA) to bypass the left main or the LAD is the gold standard, especially since it is the most important factor in improving survival and freedom from MACE in patients undergoing CABG [17]. Indeed, the use of LIMA to LAD ranks as the only class I recommendation regarding CABG conduits in the current ESC/EACTS guidelines on myocardial revascularisation [9]. It is beyond doubt that the LIMA is a durable conduit: when used as a coronary graft it is superior not only to SVGs, but also to other arterial grafts in terms of lower tendency to vasospasm and enhanced endothelial function as well as nitric oxide and prostacyclin release [18]. These features confer to it an extreme resistance to atherosclerosis [17], leading to better short-term and long-term outcomes [17–19]. Above all, the >90% ten-year patency of LIMA grafting is very noteworthy [14, 19, 20].

However, one question arises: what is the biological mechanism behind such peculiar resistance to the atherosclerotic process? The LIMA is a transition artery that combines some microscopic features of large vessels, such as the elasticity of the aorta, with others from medium-sized vessels, such as the abundance of smooth muscle cells of coronary arteries. The cross-sectional size of the LIMA measures 1.9 to 2.6 mm with a wall thickness of 180 to 430 microns [21]. The predominant layer in the LIMA is a thin tunica media, which consists of collagen and smooth muscle cells aligned circumferentially in between the elastic layers, while a muscular component is barely represented [21, 22]. These structural features are the determinants of wall stiffness [21], but also allow compliance at high pressure and, together with the small size of the vessel, help to keep a laminar blood flow, avoiding turbulence, which is a known trigger of thrombotic and atherosclerotic processes. Furthermore, the LIMA has a flow rate much more similar to that of the LAD compared with what SVGs display, resulting in a minor risk of graft thrombosis. The intima of the LIMA consists of endothelium with few fenestrations and low intercellular junction permeability, which inhibits cellular migration and prevents lipoproteins from entering the sub-endothelial space leading to intimal hyperplasia and atheroma [14]. Such endothelial cells are also rich in heparin sulphate, endothelial nitric oxide synthase (eNOS) and prostacyclin, which contribute to the antithrombotic properties, enhance endothelial homeostasis and protect from atherosclerosis [21]. The pivotal role of the eNOS in atherogenesis inhibition was demonstrated by in-vitro immunohistochemical studies conducted by He and colleagues [23]. They hypothesised a connection between the superior long-term performance of the internal mammary artery (IMA) and the higher expression level of eNOS in its endothelial cells compared with other arterial grafts. Therefore, besides supplying blood flow to the diseased coronary vessel, the LIMA graft has the peculiarity of influencing the pathophysiology of the atherosclerotic plaque.

Finally, there are very few contraindications to the use of the LIMA, such as significant disease of the subclavian artery, extreme chest deformities, emergency situations and previous chest radiotherapy [24].

## Saphenous vein graft

Since first used for CABG in the early 1960s, SVGs have extensively demonstrated the benefit of surgical revascularisation. However, their fast atherosclerotic degeneration, as compared with the native coronary arteries and with arterial grafts, raised serious concerns about their widespread use. These were confirmed by the demonstration of 15–20% probability of occlusion at 1‑year follow-up, and up to 40–50% at 10-year, with a graft attrition rate of 1–2% per year in the first six years and 4% per each subsequent year [20].

Different causes seem to be responsible for SVG failure at different time points. Within the first month from implantation, the principal mechanism involved seems to be graft thrombosis, as a direct consequence of endothelial disruption and cell loss caused by mechanical trauma during the graft harvesting, preparation, and insertion processes, even if correctly performed [21, 25]. Indeed, harvested SVGs may show large thrombogenic defects with exposed collagenous fibrils [26]. Endothelial injury is also perpetrated by haemodynamic stress on the vein wall suddenly subjected to arterial pressure and pulsatile flow [25], which stretches SVGs to a much larger diameter (ranging from 3.1 to 8.5 mm) than the LIMA [14]. However, in contrast with what is observed in the LIMA, the vein wall shows poor elastic recoil and increased stiffness [21], subsequently producing turbulence of blood flow and significant reduction of mean blood velocity [14, 20, 21]. The presence of intact venous valves may also be a predisposing factor [20, 21]. Furthermore, if not completely occlusive, thrombotic material is progressively organised into fibrotic tissue leading to smooth muscle cell migration and proliferation, which are the basis of intimal hyperplasia. Intimal hyperplasia is defined as the accumulation of smooth muscle cells and extracellular matrix (proteoglycans and type-I and type-III collagen) in the intimal compartment and represents the major cause of graft disease between one month and one year after implantation [14, 20]. These structural changes in the vein wall pave the way to the development of atherosclerosis, the predominant process of SVGs attrition in the subsequent years [14, 20]. Atherosclerotic plaques in SVGs tend to be diffuse, friable, poorly calcified and characterised by fibroatheroma that usually presents with large necrotic cores, rich in inflammatory and foam cells, and with a very thin fibrolipidic cap, compared with arterial atherosclerosis, making the plaque more susceptible to rupture [20]. It is also important to highlight that SVGs are often seriously diseased before being harvested and operators frequently neglect this. Pre-existing intimal hyperplasia due to phlebosclerosis affects up to 95% of saphenous veins after the sixth decade of life [25].

## Radial artery

The only viable strategy to avoid vein graft disease is to utilise autologous arterial grafts rather than SVGs whenever possible. Several arteries have been investigated and the radial artery (RA) is probably the most utilised. First used in 1974 by Carpentier and colleagues, the RA as coronary graft was soon abandoned due to excessive tendency toward profound vasospasm and resultant high graft failure rates [22]. The structure of the RA presents a continuous intima of endothelial cells, a single elastic lamina and a thick tunica media of smooth muscle cells, which explains its predisposition to spasm. Even though the RA shows a higher incidence of atherosclerosis compared with the LIMA [27], it seems to be provided with mechanisms that increase blood flow and prevent the development of atherosclerotic plaque [13, 20]. The endothelial cells of the RA seem to express lower levels of eNOS than those of the IMA, which is linked to lower nitric oxide release and might be related to the inferior long-term patency rate of the RA compared with the IMA [23]. The RA is one of the two arteries of the forearm and, because of its superficial and accessible location, can be easily harvested and its length usually allows all coronary territories to be reached. The Radial Artery Versus Saphenous Vein Patency Randomised Trial showed that RA grafts, similarly to LIMA grafts, are free of late graft attrition, at least at 5‑year follow-up [28]. The Radial Artery Patency Study confirmed lower functional occlusion rates of RA grafts at 1 year and significant lower frequency of complete graft occlusion at 5‑year angiographic follow-up compared with SVGs [29]. Atherosclerotic disease of the RA before harvesting may limit its use in older patients, but age should not represent an exclusion criterion per se [30]. In many catheterisation labs, the RA is unquestionably the primary vascular access for performing coronary angiographies. On this issue, there are still concerns whether the procedures of puncturing, sheath insertion and retrieval at the time of the diagnostic angiography may affect RA patency if used later for CABG [31, 32]. A known limitation is represented by possible hand circulatory disorders, ranging from claudication to resting pain or even gangrene, in case of insufficiency of the ipsilateral ulnar artery. Despite the frequent use of the Allen test to evaluate hand circulation before graft removal, around 10% of patients may still develop mild hand ischaemia after RA harvest [33]. Haemodialysis may also be considered a contraindication to RA harvest due to possible need of upper limb dialysis access [34]. As for other arterial grafts, the RA is very sensitive to competitive flow: the 2011 American College of Cardiology Foundation/American Heart Association guidelines state that the use of the RA is reasonable when grafting left-sided coronary arteries with severe stenoses (>70%) and right-sided arteries with critical stenoses (>90%) that perfuse the left ventricle (class IIb) [35].

## Gastroepiploic artery

More recently, the right gastroepiploic artery (GEA) has also been used as a coronary bypass conduit and has been recognised as a suitable and reliable alternative. The GEA is a branch of the gastroduodenal artery that runs parallel to the greater curvature of the stomach, but may present a wide range of anatomical variants. The proximal portion of the GEA usually measures 2.5 to 3 mm in diameter and in most cases remains greater than 1.5 mm in the middle of the greater curvature. Suma and colleagues, in their experience with GEA grafting, describe a 5-year patency rate of 85.5% [36], implying a significant durability of the GEA graft, which seems to have a low incidence of atherosclerosis and sufficient flow capacity to be used as CABG [36, 37]. The in situ GEA easily reaches the inferior wall of the myocardium without stretching and appears particularly suitable for anastomosis to the posterior descending artery or to the proximal right coronary artery [38]. However, harvest of the GEA usually requires a trans-diaphragmatic approach or extra-abdominal incision, which may increase operative time and expose the patient to additional complications. Laparoscopic harvesting may be possible, but is reserved for selected cases and is not part of common clinical practice. Furthermore, performing an angiography of an in situ GEA is definitely more challenging than any other graft due to its anatomical location. Contraindications include disease of the descending aorta and not severe narrowing of a dominant right coronary artery [12, 24].

## Right internal mammary artery

The demonstrated successful performance and durability of the LIMA graft led many surgeons to believe that the same benefits could also apply to the right internal mammary artery (RIMA). Indeed, in a vast meta-analysis of 27 observational studies by Weiss et al., a significant increase in long-term survival was reported in patients undergoing bilateral mammary artery (BIMA) grafting when compared with the patients undergoing lone LIMA grafting [39]. Nevertheless no randomised controlled trials have been included in such meta-analyses and it remains unclear whether this advantage is directly dependent on the use of the BIMA or should be simply ascribed to the already demonstrated benefits of using arterial conduits. As a matter of fact, BIMA grafting represents only 4–12% of all CABG procedures over the more traditional use of the LIMA with SVGs [39]. Moreover, recent evidence suggests that BIMA grafting rates are higher in Europe than in the USA (25 vs 10%) [40]. Concerns about the use of BIMA grafting are mostly connected to a potential increase of perioperative morbidity and mortality (possibly depending on increased duration of surgical operation) and a higher probability of developing deep sternal wound infections or dehiscence [41]. Deep sternal wound infections can be a serious, life-threatening condition due to the damaging of the sternal microcirculation occurring during the harvesting process. In fact, simultaneous harvesting of both IMAs may impair sternal perfusion and compromise the wound healing process, especially in patients suffering from diabetes mellitus, chronic obstructive pulmonary disease or obesity [41]. Careful patient selection and harvesting skeletonised IMAs, which seems to preserve sternal microcirculation over the traditional pedicled technique, may minimise this complication [39, 42]. The Arterial Revascularisation Trial (ART) is an ongoing randomised trial comparing BIMA with single IMA CABG: initial results from this study have not shown significant difference between the two groups in terms of survival or MACE incidence at 30-day or at 1‑year. On the other hand, sternal wall reconstruction was more frequently required in the BIMA group (1.9% vs 0.6%) [41].

## Grafting and stenting: hybrid coronary artery revascularisation

An appealing option for the treatment of multi-vessel CAD (MVCAD) is represented by hybrid coronary artery revascularisation (HCAR). This technique combines the long-term benefits of LIMA grafting on the LAD and the minimal invasiveness of PCI on non-LAD territories. HCAR aims to minimise surgical-related impact on quality of life by performing a LIMA-LAD graft during off-pump mini-sternotomy, followed by implantation of DES in non-LAD lesions. This approach is typically destined for patients at high risk of MACE, affected by MVCAD with a complex LAD lesion and non-complex non-LAD lesions, who otherwise would more likely undergo traditional CABG [43]. Therefore, HCAR normally allows a quicker recovery after surgery, generating less perioperative morbidity and a lower incidence of adverse events connected to cardiopulmonary bypass and full sternotomy [43, 44]. However, according to the latest ESC/EACTS guidelines on myocardial revascularisation [9], the treatment of choice for MVCAD with important and/or complex stenosis of the LAD, and with acceptable surgical risk, remains traditional CABG.

## Discussion

The choice of conduit is of outmost importance for the long-term success of CABG. The benefits of using the LIMA to bypass the LAD have been extensively ascertained and explain the pivotal role that this graft nowadays holds in common surgical practice. Although the use of the LIMA to the LAD is essential, MACE-free survival after CABG also correlates with the completeness of myocardial revascularisation [10] and, for this reason, the choice for the second-best graft is a constant source of debate (Table 1 and 2). Some studies reported a better MACE-free survival rate with the use of the RIMA over the RA as second arterial graft [45, 46], while others support the RA over the RIMA, which is considered to be excessively prone to postoperative complications in certain groups of patients [41, 45]. The GEA gave excellent long-term results as third arterial graft [47], but despite its good patency rates, it is burdened by a challenging harvesting process and concerns about complications. The more widespread use of SVGs contrasts with the well-known long-term efficacy of multiple arterial grafting. Indeed, several clinical trials suggest that arterial revascularisation should be encouraged [13, 16, 22, 29, 34, 45, 46, 48–51]. In Table 3 we report the latest recommendations of Society of Thoracic Surgeons guidelines on arterial conduit choice [11] and in Table 4 evidence of patency in different conduits from recent studies [28, 29, 36, 52–54]. Locker et al. [13] describe a linear correlation between late graft patency and survival: the absence of arterial grafts was recognised as an independent predictor of late death, which relates to their excellent patency rates compared with SVGs, assessed around 95.5% at 12 weeks and 88.9% at 12 months for RA and similar for other arterial conduits [18]. In spite of its demonstrated benefits, multiple arterial revascularisation is not routinely used and struggles to establish itself as a procedure of choice. The reasons behind the low use of arteries as second graft lie in the concerns of increased technical difficulties and higher risk of postoperative complications. Furthermore, arterial grafts can be severely damaged by native competitive flow: surgical revascularisation of angiographically moderate coronary stenosis is associated with a higher risk of graft dysfunction in arterial grafts compared with SVGs. [12, 55, 56]. Therefore, before choosing an arterial revascularisation strategy, careful functional characterisation by fractional flow reserve of a borderline stenosis should be considered.

**Table 1:** Specific factors influencing the choice of the second-best graft conduit

Conduit	Advantages	Main concerns	Contraindications
SVG	Short operative time Basic skills required	High attrition rate Pre-existing vein disease Easily damaged during harvesting	Previous vein sclerotherapy or vein removal Ongoing (or previous) phlebitis
RA	Easy harvesting Long and versatile graft	Post-operative hand ischaemia Tendency to spasm Previous cannulation	Target stenosis <70% Need of upper limb haemodialysis
GEA	In situ use for inferior wall	Challenging harvesting technique Tendency to spasm	Target stenosis <90% Disease of descending aorta Abdominal surgery impairment
RIMA	High durability	Deep sternal wound infection Time consuming procedure	Target stenosis <70% Previous chest radiotherapy Right subclavian artery disease Extreme chest deformity

*SVG* saphenous vein graft, *RA* radial artery, *GEA* right gastro-epiploic artery, *RIMA* right internal mammary artery

**Table 2:** Quantification of aspects influencing the choice of the second-best graft conduit

Features	SVG	RIMA	RA	GEA
Attrition rate	++++	+	++	++
Tendency to vasospasm	+	++	++++	+++
Failure if target stenosis <70%	+	++	++	+++
Operative time	+	+++	++	+++
Resistance to harvesting trauma	+	+++	++	++
Nitric oxide and prostacyclin release	–	+++	+	+
Frequency of structural or functional abnormalities at harvesting	++	–	+	+
Flow reserve	–	+++	++	+++

*SVG* saphenous vein graft, *RA* radial artery, *GEA* right gastro-epiploic artery, *RIMA* right internal mammary artery

**Table 3:** The 2015 Society of Thoracic Surgeons (STS) guidelines on arterial conduits for CABG

Recommendations	COR	LOE
The IMA should be used to bypass the LAD artery when bypass of the LAD is indicated	I	B
As an adjunct to LIMA, a second arterial graft (RIMA or RA) should be considered in appropriate patients	IIa	B
Use of BIMA should be considered in patients who do not have an excessive risk of sternal complications	IIa	B
As an adjunct to LIMA to LAD (or in patients with inadequate LIMA grafts), use of a RA graft is reasonable when grafting coronary targets with severe stenoses	IIa	B
When RA grafts are used, it is reasonable to use pharmacologic agents to reduce acute intraoperative and perioperative spasm	IIa	C
Use of arterial grafts (specific targets, number, and type) should be a part of the discussion of the heart team in determining the optimal approach for each patient	I	C

*COR* class of recommendation, *LOE* level of evidence, *ITA* internal thoracic artery, *LAD* left anterior descending artery, *LIMA* left internal mammary artery, *RIMA* right internal mammary artery, *BIMA* bilateral internal mammary artery, *RA* radial artery

**Table 4:** Patency data from recent comparative studies among arterial conduits for CABG

First Author	Year	Patients	Follow-up (years)	SVG patency (%)	RA patency (%)	RIMA patency (%)	RGEA patency (%)
Deb [29]	2012	269	7.7 ± 1.5	81.4	91.1	–	–
Goldman [52]	2011	733	1	89	89	–	–
Suma [36]	2007	124	5–17	68	–	–	87
Hayward [53]	2011	214	5.5	84.7	92.2	–	–
Collins [28]	2011	104	5	77	90	–	–
Hwang [54]	2013	566	5	–	–	92.7	89.6

*SVG* saphenous vein graft, *RA* radial artery, *RIMA* right internal mammary artery, *RGEA* right gastroepiploic artery

## Conclusions

The therapeutic use of LIMA in patients with MVCAD is the best performing arterial-coronary conduit compared with other arterial or venous grafts, measured both in patency and in clinical outcome. Moreover, because of its distinct physiological properties, it seems to have a healing effect on distal atherosclerosis, although this effect needs further elaboration. Furthermore we described the superiority of different arterial grafts compared with venous grafts, although the latter are still the most frequently used in many centres. If the graft choice as strategic issue is incorporated into heart team discussions more appropriate use of arterial grafts can be expected. What is interesting is the upcoming hybrid strategy after decades of a dichotomic evaluation and comparison of CABG and PCI in patients with triple-vessel disease. Contemporary decision-making in coronary revascularisation will be more than a simple choice between knife or balloon, and an elaborate choice of conduit and strategy is part of that.
